# A Novel Semi-Supervised Electronic Nose Learning Technique: M-Training

**DOI:** 10.3390/s16030370

**Published:** 2016-03-14

**Authors:** Pengfei Jia, Tailai Huang, Shukai Duan, Lingpu Ge, Jia Yan, Lidan Wang

**Affiliations:** College of Electronic and Information Engineering, Southwest University, Chongqing 400715, China; jiapengfei200609@126.com (P.J.); 18580465830@163.com (T.H.); gelingpu@126.com (L.G.); yanjia119@163.com (J.Y.); ldwang@swu.edu.cn (L.W.)

**Keywords:** electronic nose, semi-supervised learning, unlabeled samples, indoor pollution gas

## Abstract

When an electronic nose (E-nose) is used to distinguish different kinds of gases, the label information of the target gas could be lost due to some fault of the operators or some other reason, although this is not expected. Another fact is that the cost of getting the labeled samples is usually higher than for unlabeled ones. In most cases, the classification accuracy of an E-nose trained using labeled samples is higher than that of the E-nose trained by unlabeled ones, so gases without label information should not be used to train an E-nose, however, this wastes resources and can even delay the progress of research. In this work a novel multi-class semi-supervised learning technique called M-training is proposed to train E-noses with both labeled and unlabeled samples. We employ M-training to train the E-nose which is used to distinguish three indoor pollutant gases (benzene, toluene and formaldehyde). Data processing results prove that the classification accuracy of E-nose trained by semi-supervised techniques (tri-training and M-training) is higher than that of an E-nose trained only with labeled samples, and the performance of M-training is better than that of tri-training because more base classifiers can be employed by M-training.

## 1. Introduction

An electronic nose (E-nose) is a device composed of a gas sensor array and an artificial intelligence algorithm. They are effective in dealing with odor analysis problems [1,2,3], and have been introduced to many fields such as environmental monitoring [4,5], food engineering [6,7,8], disease diagnosis [9,10,11,12], explosives detection [13] and spaceflight applications [14].

Most of the time during a person’s life is spent indoors, so it is significant to monitor changes in indoor gas composition, and it is necessary for people’s health to detect the indoor pollutant gases as early as possible. Consequently, there has been a resurgence of interest in developing measurement techniques for air quality monitoring. Our previous work has proved that E-noses are an effective way to classify indoor pollutant gases [15,16].

To study the patterns of different indoor pollutant gases, many sampling experiments must be done on each gas. In the past, we only processed labeled data by feature extraction methods [17,18], however, in actual experiments, the numbers of collected unlabeled samples are often far greater than that of the labeled samples, and they are easier to obtain while the cost of getting the labeled samples is usually higher than for unlabeled ones. On the other hand, in the sampling experiments, there can be unexpected mistakes such as the paper label identifying the target gas which is pasted on the gas bag is lost, the label information is not written down because of a fault of operators which will lead to the loss of the sample label, which all causes a certain amount of waste of the number of experimental samples. Although the classification accuracy of E-noses trained by labeled samples is usually higher than that of devices trained with unlabeled samples, it is often difficult to obtain sufficient labeled samples. What’s more, there is a lot of hidden information in the unlabeled samples. Therefore, researchers have put forward algorithms to train E-noses with labeled sample as well as make full use of available unlabeled samples.

To make full use of unlabeled samples, researchers have proposed various methods in the past. These methods can be divided into three categories: (1) *Active learning*: this is a learning paradigm that requires users’ (or some other information source) interaction to provide the responses of new data points [19,20]; (2) *Transfer learning*: these are methods that focus on applying the knowledge learned from related, but different tasks to solve the target task [21,22,23]. They usually require sufficient labeled data to acquire accurate knowledge; (3) *Semi-supervised learning (SSL)*: these techniques aim at learning an inductive rule or try to accurately determine the label of the data from a small amount of labeled data with the help of a large amount of unlabeled data [24,25,26]. For its ability to solve classification and regression problems by learning from a set of labeled data and unlabeled samples, this last approach has been widely adopted in various application domains such as hand-writing recognition [27] and bioinformatics [28].

In 2012, De Vi *et al.* applied a semi-supervised boosting algorithm to an artificial olfaction classification problem and proposed a novel SSL-based algorithm for an air pollution monitoring data set [29]. This work can be thought as the first time of SSL was adopted in E-nose research. Liu *et al.* also proposed a domain adaptation technique which can be seen as a SSL technique in 2014, and this technique was adopted to eliminate the E-nose signal drift [30].

Tri-training is a SSL techniques [31] which doesn’t require sufficient and redundant samples, nor does it require the use of different supervised learning algorithms. Inspired by tri-training, a novel multi-class SSL technique which is called as M-training is proposed in this paper to train E-noses with both labeled samples and unlabeled samples. The rest of this paper is organized as follows: Section 2 introduces the E-nose system and gas sampling experiments of this paper; Section 3 presents the theory of M-training technique; Section 4 describes the results of M-training when it is used to train the E-nose classifier for predicting the classes of target pollutant gases. Finally, we draw the conclusions of this paper in Section 5.

## 2. E-Nose System and Experiments

### 2.1. Target Gas and Experimental Setup

Three common kinds of indoor pollutant gas including benzene (C_6_H_6_), toluene (C_7_H_8_) and formaldehyde (CH_2_O) were the target gases which will be distinguished by the E-nose. The sensor array of the E-nose presented in this paper contains five sensors: three metal oxide semi-conductor gas sensors (TGS2620, TGS2602 and TGS2201 purchased from Figaro Company, Osaka, Japan). The TGS 2201 has two outputs defined as TGS 2201A and TGS 2201B), one humidity sensor and one temperature sensor. The sensitive characteristics of the three gas sensors is shown in Table 1.

**Figure 1:**
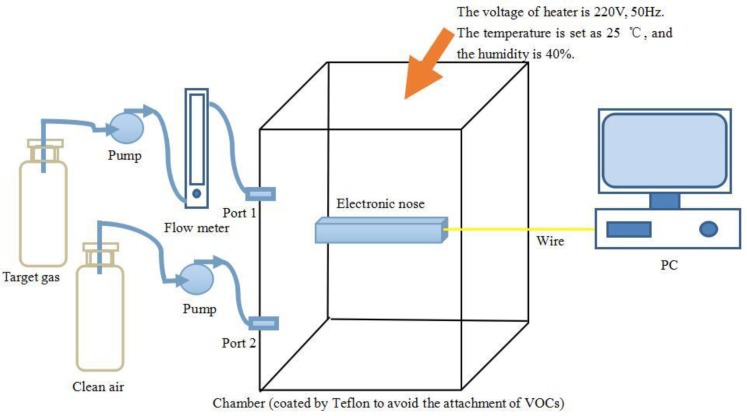
Schematic diagram of the experimental system.

**Figure 2:**
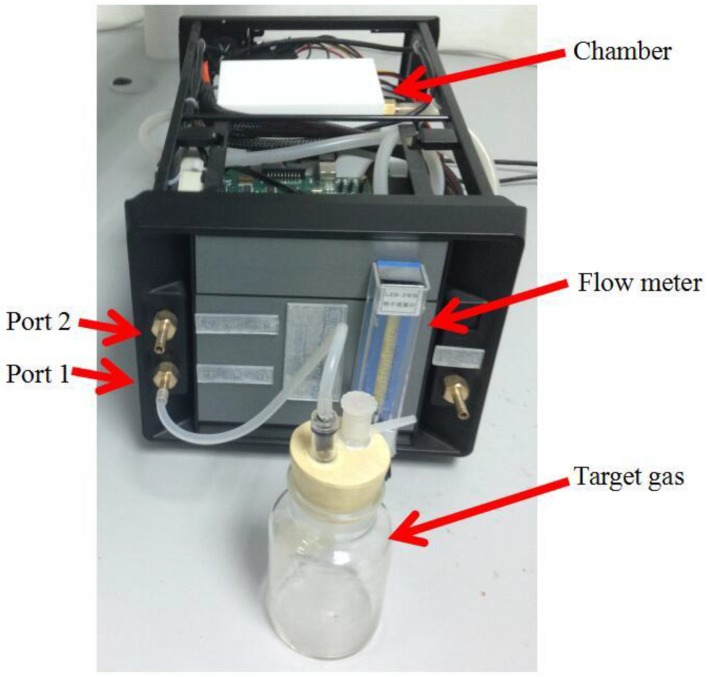
Image of the experimental setup.

A 12-bit analog-digital converter (A/D) is used as interface between the sensor array and a field programmable gate array (FPGA) processor. The A/D converts analog signals from sensor array into digital signal, and the sampling frequency is set as 1 Hz. As is shown in Figure 1, the experimental platform mainly consists of the E-nose system, a PC, a temperature-humidity controlled chamber (coated with Teflon to avoid the attachment of VOCs), a flow meter and an air pump. There are two ports on the sidewall of the chamber, and the target gas and the clean air are put into the chamber through ports 1 and 2, respectively. Data collected from the sensor array can be saved on a PC through a joint test action group (JTAG) port and its related software. An image of the experimental setup is shown in Figure 2.

### 2.2. Sampling Experiments and Data Pre-Processing

Before sampling experiments, we firstly set the temperature and humidity of the chamber as 25 °C and 40%. Then we can begin the gas sampling experiments, and one single sampling experiment incorporates three steps:

Step 1: All sensors are exposed to clean air for 2 min to obtain the baseline;

Step 2: Target gas is imported into the chamber for 4 min;

Step 3: The array of sensors is exposed to clean air for 9 min again to wash the sensors and make them recover their baseline signal.

**Figure 3:**
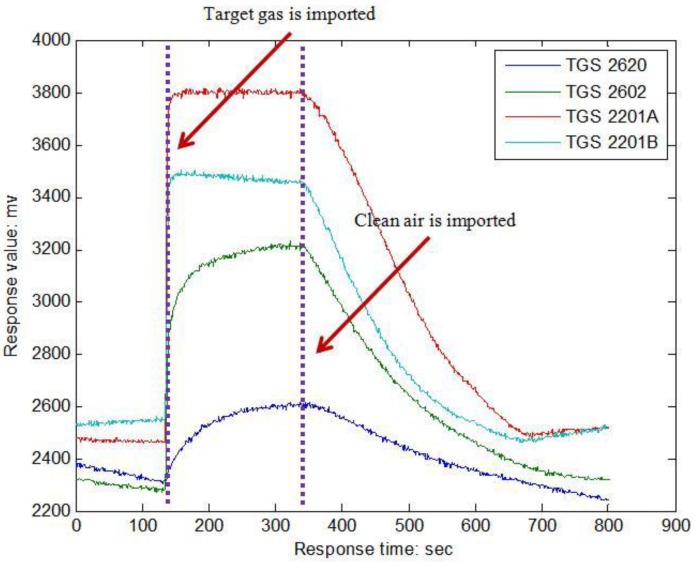
Response of the sensors array.

Figure 3 illustrates the response of sensors when formaldehyde is introduced into the chamber. One can see that each response curve rises obviously from the third minute when the target gas begins to pass over the sensor array, and recovers to baseline after the seventh minute when clean air is imported to wash the sensors.

To get the real concentration of gas in the chamber, we extract each gas from the chamber and import it into a gas bag. Then a spectrophotometric method is employed to get the concentration of formaldehyde, and the concentration of benzene and toluene are determined by gas chromatography (GC). For each gas, there are 12, 11 and 21 concentration points, respectively, and 12 sampling experiments are made on each concentration point. The real concentration and the numbers of samples of the three kinds of gas are shown in Table 2.

Then the maximum value of the steady-state response of sensors is extracted to create the feature matrix of the E-nose. There are 528 samples in this matrix and the dimension of each sample is 4. We randomly select 75% of the samples of each gas to establish the training data set, and the rest are used as the test data set. Detailed information is shown in Table 3.

## 3. M-Training Technique

As a SSL technique, M-training retains the advantages of tri-training, while, more base classifiers can be employed by M-training which gives it have more opportunity to learn and obtain knowledge from the unlabeled samples.

Let *L* denote the labeled sample set with size |*L*| and *U* denote the unlabeled sample set with size |*U*|. There are *M* base classifiers in M-training, denoted as $c_{i},i=1,2,\cdots ,M$, where *M* is a positive integer, and *M* ≥ 3. M-training will degenerate to tri-training when *M* is set as 3. These base classifiers have been trained by the samples from set *L*. During the learning process of M-training, each *c_i_* will be the main classifier in a cycle, meanwhile, the other classifiers are employed to predict the class label of samples from *U* (for simply, these classifiers are denoted as $C_{i},i=1,2,\cdots ,M$). Whether one sample of set *U* will be used to train the main classifier *c_i_* combining with set *L* depends on the degree of agreements (made by classifiers of *C_i_*) on its labeling, namely, if the classifiers of *C_i_* voting for a particular label exceeds a threshold θ, then this sample along with its label (predicted by *C_i_*) will be used to refine the main classifier *c_i_* combining with set *L*.

In the M-training technique, the misclassification of unlabeled samples is unavoidable, so *c_i_* will receive noisy samples from time to time. Fortunately, even in the worst case, the increase in classification noise rate can be compensated if the amount of newly labeled samples is sufficient and meet certain conditions. These conditions are introduced as follows:

Inspired by Goldman *et al.* [32], the finding of Angluin *et al.* [33] is employed. Suppose there is a training data set containing *m* samples, and the noise rate is *η*, then the worst case error rate ξ of the classifier satisfies Equation (1):

(1) $$m=\frac{\sigma }{\xi^{2}{\left(1-2\eta \right)}^{2}}$$

where σ is a constant, then Equation (1) can be reformulated as Equation (2):

(2) $$u=\frac{\sigma }{\xi^{2}}=m{\left(1-2\eta \right)}^{2}$$

In each round of M-training, *C_i_* chooses samples in *U* to label for *c_i_*. The amount and the concrete unlabeled samples chosen to label would be different in different rounds because *c_i_* is refined in each round. We denote by *L_i_*(*t*) and *L_i_*(*t* – 1) the set of samples which are labeled by *C_i_* for *c_i_* in round *t* and round *t* – 1, respectively. Then the training data set for *c_i_* in round *t* and *t* – 1 can be expressed as $\left|L\cup L_{i}(t)\right|$ and $\left|L\cup L_{i}(t\;-\;1)\right|$, respectively. It should be noted that *L_i_*(*t* – 1) will be regarded as the unlabeled data and put back to *U* during round *t*.

Let *η_L_* denote the classification noise rate of *L*, so the number of mislabeled samples in *L* is *η_L_*|*L*|. Let *e_i_*(*t*) be the upper bound of the classification error rate of *C_i_* in round *t*. Assuming there are *n* samples which are labeled by *C_i_*, and among these samples, *C_i_* makes the correct classification on *n*’ samples, then *e_i_*(*t*) can be estimated as (*n – n*’)/*n*. Thus, the number of mislabeled samples in *L_i_*(*t*) is $e_{i}(t)\left|L_{i}(t)\right|$. Therefore the classification noise rate in round *t* is:

(3) $$\eta_{i}(t)=\frac{\eta_{L}\left|L\right|+e_{i}(t)\left|L_{i}(t)\right|}{\left|L\cup L_{i}(t)\right|}$$

Thus, Equation (2) can be computed as:

(4) $$\begin{array}{ll} u_{i}(t) & =m_{i}(t){\left(1-2\eta_{i}(t)\right)}^{2}\\ & =\left|L\cup L_{i}(t)(1-2\frac{\eta_{L}\left|L\right|+e_{i}(t)\left|L_{i}(t)\right|}{\left|L\cup L_{i}(t)\right|})\right| \end{array}$$

Similarly, $u_{i}(t-1)$ can be computed by Equation (5):

(5) $$\begin{array}{ll} u_{i}(t-1) & =m_{i}(t-1){\left(1-2\eta_{i}(t-1)\right)}^{2}\\ = & \left|L\cup L_{i}(t-1)(1-2\frac{\eta_{L}\left|L\right|+e_{i}(t-1)\left|L_{i}(t-1)\right|}{\left|L\cup L_{i}(t-1)\right|})\right| \end{array}$$

If we want $e_{i}(t)<e_{i}(t-1)$, then $u_{i}(t)>u_{i}(t-1)$ according to Equation (2), which means that the performance of *c_i_* can be improved through utilizing *L_i_*(*t*) in its training. This condition can be expressed as Equation (6):

(6) $$\begin{array}{l} \left|L\cup L_{i}(t)(1-2\frac{\eta_{L}\left|L\right|+e_{i}(t)\left|L_{i}(t)\right|}{\left|L\cup L_{i}(t)\right|})\right|>\\ \left|L\cup L_{i}(t-1)(1-2\frac{\eta_{L}\left|L\right|+e_{i}(t-1)\left|L_{i}(t-1)\right|}{\left|L\cup L_{i}(t-1)\right|})\right| \end{array}$$

Considering that $\eta_{L}$ can be very small and assuming $0\leq e_{i}(t-1),e_{i}(t)\leq 0.5$, then the first part on the left hand of Equation (6) is bigger than its correspondence on the right hand if $\left|L_{i}(t-1)\right|<\left|L_{i}(t)\right|$, and the second part on the left hand is bigger than its correspondence on the right hand if $e_{i}(t)\left|L_{i}(t)\right|<e_{i}(t-1)\left|L_{i}(t-1)\right|$. These restrictions can be expressed into the condition shown in Equation (7), and this condition is employed by M-training to decide whether one unlabeled sample could be labeled for *c_i_*:

(7) $$0<\frac{e_{i}(t)}{e_{i}(t-1)}<\frac{\left|L_{i}(t-1)\right|}{\left|L_{i}(t)\right|}<1$$

Note that $e_{i}(t)\left|L_{i}(t)\right|$ may still be less than $e_{i}(t-1)\left|L_{i}(t-1)\right|$ even if $e_{i}(t)<e_{i}(t-1)$ and $\left|L_{i}(t-1)\right|<\left|L_{i}(t)\right|$ due to the fact that $\left|L_{i}(t)\right|$ may be much bigger than $\left|L_{i}(t-1)\right|$. When this happens, a sub-sampling method presented in paper [31] is employed, and the detail operation is shown as follows: in some cases $L_{i}(t)$ could be randomly sub-sampled such that $e_{i}(t)\left|L_{i}(t)\right|<e_{i}(t-1)\left|L_{i}(t-1)\right|$. Given $e_{i}(t)$, $e_{i}(t-1)$ and $\left|L_{i}(t-1)\right|$, let integer $s_{i}$ denote the size of $L_{i}(t)$ after sub-sampling, then if Equation (8) holds, $e_{i}(t)\left|L_{i}(t)\right|<e_{i}(t-1)\left|L_{i}(t-1)\right|$ will be satisfied:

(8) $$s_{i}=\left\lceil \frac{e_{i}(t-1)\left|L_{i}(t-1)\right|}{e_{i}(t)}-1\right\rceil$$

where $L_{i}(t-1)$ should satisfy Equation (9) such that the size of $L_{i}(t)$ after sub-sampling is still bigger than $\left|L_{i}(t-1)\right|$:

(9) $$\left|L_{i}(t-1)\right|>\frac{e_{i}(t)}{e_{i}(t-1)-e_{i}(t)}$$

It is noteworthy that the initial base classifiers should be diverse because if all classifiers are identical, then for any *c_i_*, the unlabeled samples labeled by classifier of *C_i_* will be the same as *c_i_*. To achieve the diversity of the base classifiers, each base classifier just randomly employ 75% of set *L* as its initial training data set, and the training data set of each classifier will be different via this way.

Finally, the process of M-training can be listed as follows:

Step (a): Prepare data set *L*, *U* and the test data set for E-nose; set the value of *M* and *θ*.

Step (b): Train each base classifier *c_i_* of M-training with the initial training data set *L_i_* generated randomly from set *L*.

Step (c): Gain the initial classification accuracy of set *L* and the initial classification accuracy of the test data set. Simple voting technique is employed to determine the predict label of one sample, and all base classifiers of M-training are used to predict the gas during this step.

Step (d): Repeat the following process until none of $c_{i}$, i=1,2,⋯,M changes:

(d.1) Compute $e_{i}(t)$, as it has been introduced that $e_{i}(t)=\frac{n_{i}(t)-n_{i}\prime (t)}{n_{i}(t)}$, where $n_{i}(t)$ means the samples of set *U* labeled by $C_{i}$ in round t, and $n_{i}\prime (t)$ is the samples of set *U* labeled correctly by *C_i_*. However it is impossible to estimate the classification error on the unlabeled samples, and only set *L* is available, heuristically based on the assumption that the unlabeled samples hold the same distribution as that held by the samples of set *U*;

(d.2) If $e_{i}(t)<e_{i}(t-1)$, any sample x of set *U* will be used to generate set $L_{i}(t)$ if the agreement of labeling this sample made by classifiers in $C_{i}$ exceeds θ;

(d.3) If $\left|L_{i}(t-1)\right|<\left|L_{i}(t)\right|$, then there will be two cases: case (1) $e_{i}(t)\left|L_{i}(t)\right|<e_{i}(t-1)\left|L_{i}(t-1)\right|$, classifier $c_{i}$ will be refined by $L_{i}\cup L_{i}(t)$, and $L_{i}(t-1)=\left\lfloor \frac{e_{i}(t)}{e_{i}(t)-e_{i}(t-1)}+1\right\rfloor$, if $L_{i}(t-1)=0$; case (2) $\left|L_{i}(t-1)\right|>\frac{e_{i}(t)}{e_{i}(t-1)-e_{i}(t)}$, then $\left|L_{i}(t)\right|-s_{i}$ samples of $L_{i}(t)$ will be removed, where $s_{i}$ is computed by Equation (8), then $c_{i}$ will be refined.

Step (e): Obtain the final classification accuracy of set *L* and the final classification accuracy of the test data set, and the computation process is the same as step (c).

## 4. Results and Discussion

The first task of this section is to decide which classifier can be used as the base classifier of M-training. Partial least square discriminant analysis (PLS-DA) [34], radial basis function neural network (RBFNN) [35] and support vector machine (SVM) [36,37] are considered in this paper. The leave-one-out technique (LOO) is used to train and test the three classifiers. Classification accuracy of the training data set and test data set is set to evaluate the performance of the three classifiers. To make sure every classifier achieves its best working state, an enhanced quantum-behaved particle swarm optimization (EQPSO) [38] is used to optimize the parameters. Each program is repeated for 10 times among which the best result will be the final result of each classifier. The results are shown in Table 4.

It is clear that the classification accuracy of SVM has the highest accuracy rate of all classifiers, so SVM is selected as the base classifier for M-training. The value of parameters in these three methods are set as follows: the number of latent variables of PLS-DA is 5; the goal MSE and the spread factor of RBFNN are 0.4329 and 0.0176, respectively; RBF function is employed as the kernel of SVM and its value is 0.2749, while the value of the penalty factor of SVM is 0.4848.

**Figure 4:**
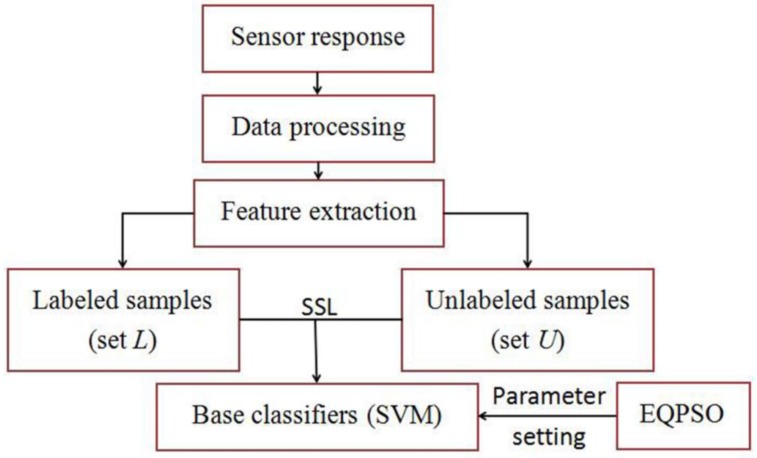
Flow chart of SSL process.

Then tri-training and M-training with a different number of base classifiers are employed to refine the classifier of our E-nose. The flow chart of SSL process is shown as Figure 4. Half of the training data set is defined as data set *L* which is used to train the base classifiers, and the rest of the training data set are set as set *U* which is used to refine the base classifiers, namely, the unlabeled rate is 50%. The threshold *θ* of M-training is set as 23. The classification results of both methods are shown in Table 5.

As one can see, the M-training results are better than those of tri-training. The reason is analyzed as follows: suppose there is a point in set *U* (whose real label is 1). There are three classifiers in tri-training, this point will not be considered to train classifier 1 if classifier 2 predicts the label of this point is 1 and classifier 3 predicts its label is 2. Meanwhile, suppose there are four classifiers in M-training, and classifier 2 and classifier 3 make the same classification as the corresponding classifier in tri-training, then this point will be considered to refine classifier 1 if classifier 4 predicts the label is 1, so M-training has more opportunity to refine its base classifiers, and this ensures that the E-nose has more opportunity to learn knowledge from the unlabeled points.

It can also be found from Table 5 that the classification results of M-training with four base classifiers are the same as M-training with five or six base classifiers. Although more base classifiers means more opportunities to learn from the unlabeled samples, the knowledge provided by unlabeled samples is limited when the set of unlabeled samples is determined. One can enlarge the size of unlabeled set to make the classification accuracy of E-nose more ideal.

The highest classification accuracy in Table 5 is 96.97% obtained by M-training when four base classifiers are trained by *L* and refined by *U* (the unlabeled rate is 50%). To study how much potential knowledge has been recovered from the unlabeled samples, we set another training process during which there are four classifiers (SVM) and they are trained by the whole training data set (*L* + *U*). The final classifier result is decided by simple voting, and its corresponding classification accuracy of the test data set is 98.48%. We can find this result is much higher than 74.24% (obtained when the four base classifiers of M-training are just trained by set *L*) and is just little higher than 96.97%. This comparison indicates that much useful knowledge has been found by the E-nose in the unlabeled data with the help of M-training.

Finally, the performance of M-training (four base classifiers) with different unlabeled rates (75%, 50% and 25%) of the training data set is researched. An introduction about the amount of samples in each data set is given in Table 6.

**Figure 5:**
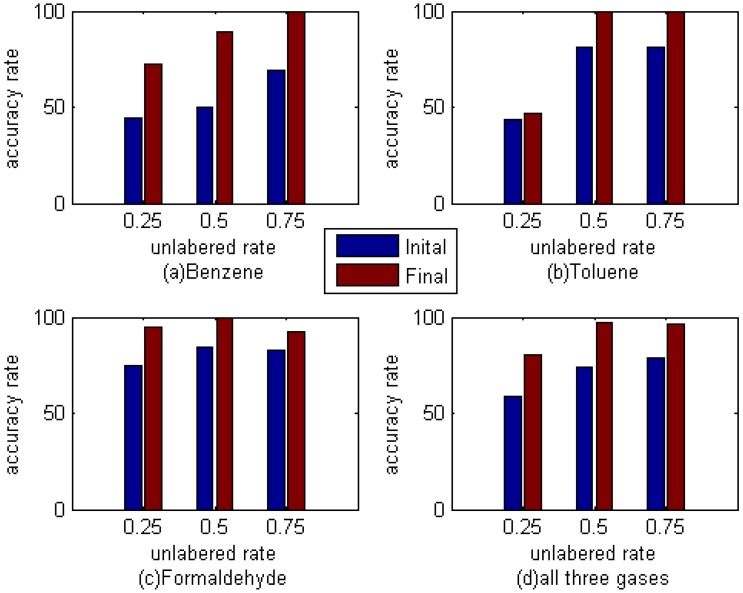
Classification accuracy of different gas in the test data set. (**a**), (**b**) and (**c**) show the classification accuracy of benzene, toluene and formaldehyde, respectively, and (**d**) shows the classification accuracy of all gas. In each figure, the accuracy is improved with the help of M-training, and the improvement is most obvious when the unlabeled rate is 50%.

Table 7, Table 8 and Table 9 list the results of the M-training technique with different unlabeled sample rates, and it is clear that the unlabeled samples can improve the classification accuracy of the test data set no matter what the unlabeled rate is. Table 10 lists the amount of samples in each *c_i_* with different unlabeled rate, and one can find that more samples are used to train the base classifier when M-training is adopted to train and refine E-nose. Figure 5 shows the classification accuracy of different gas in the test data set. As can be seen, the recognition rate of these three kinds of gas, whether a single case or all three kinds of gas, has improved in varying degrees. And on the whole, the effect is most obvious when the unlabeled rate is 50%. Whether this is the best proportion is still need to be further verified, but it can be determined that M-training technique can indeed improve the accuracy rate of E-nose to these three kinds of gas.

## 5. Conclusions

In this paper, we propose a novel algorithm that not only uses labeled samples to train an E-nose, but also can correct the trained algorithm model by using unlabeled samples. In the past, researchers trained E-noses using labeled samples, discarding the unlabeled samples, which wastes a large number of samples because unlabeled samples also contain useful information. To make good use of the unlabeled gas samples, a proposed E-nose semi-supervised learning technique (M-training) is used to improve the classification accuracy of the E-nose in predicting three common indoor pollutant gases (benzene, toluene and formaldehyde), during which the classifier is trained with labeled samples and refined by unlabeled samples. The data processing results of tri-training and M-training with different numbers of base classifiers prove that the classification accuracy of the E-nose is been improved when unlabeled samples are used to refine the E-nose by these semi-supervised methods.

In some cases, there are more opportunities for M-training to learn knowledge from the unlabeled samples if it contains more base classifiers, but the accuracy of the classification is unlikely to reach 100% even if the number of base classifiers approaches infinity. There is a reason that the knowledge provided by the unlabeled samples is limited as long as the set of unlabeled samples is determined, but it can enlarge the size of unlabeled set to make the classification accuracy of an E-nose more ideal when M-training is used to train an E-nose. All results make it clear that M-training is an effective multi-class semi-supervised technique for E-noses used to distinguish benzene, toluene and formaldehyde.

## Figures and Tables

**Table 1:** Main sensitive characteristics of gas sensors.

Sensors	Main Sensitive Characteristics
TGS2620	Carbon monoxide, ethanol, methane, isobutane, VOCs
TGS2602	Ammonia, formaldehyde, toluene, ethanol, hepatic gas, VOCs
TGS2201	Carbon monoxide, nitric oxide, nitrogen dioxide

Note: The response of these three sensors is non-specific. Table 1 lists their main sensitive gas, but they are also sensitive to other gases.

**Table 2:** Concentration of the target gas.

Gas	Concentration Range (ppm)	Number of Samples
Benzene	[0.1721, 0.7056]	144 (12 × 12)
Toluene	[0.0668, 0.1425]	132 (12 × 11)
Formaldehyde	[0.0565, 1.2856]	252 (12 × 21)

**Table 3:** Amount of samples in training set and test set.

Gas	Training Set	Test Set
Benzene	108	36
Toluene	100	32
Formaldehyde	188	64
All-3	396	132

**Table 4:** Classification accuracy of different classifiers (%).

	PLS-DA	RBFNN	SVM
Classification accuracy of training data set	87.88	92.03	96.59
Classification accuracy of test data set	87.88	89.02	96.21

**Table 5:** Performance of tri-training and M-training with different number of base classifiers (%).

	Classification Accuracy (Initial)	Classification Accuracy (Final)	Impro
Tri-training	73.48	91.67	24.76
M-training (4 base classifiers)	74.24	96.97	30.62
M-training (5 base classifiers)	74.24	96.97	30.62
M-training (6 base classifiers)	74.24	96.97	30.62

Note: Impro = (final accuracy-initial accuracy)/initial accuracy; The initial classification accuracy of the test data set is obtained when just set *L* is used to train the base classifiers, and the final classification accuracy is obtained when set *U* is adopted to refine the base classifiers which have been trained by set *L*.

**Table 6:** Amount of samples in each data set.

	Amount of Samples in Training Data Set	Amount of Samples in *L* 25%/50%/75%	Amount of Samples in *U* 25%/50%/75%	Amount of Samples in Test Data Set
Benzene	108	27/54/81	81/54/27	36
Toluene	100	25/50/75	75/50/25	32
Formaldehyde	188	47/94/141	141/94/47	64
All-3	396	99/198/297	297/198/99	132

Note: 25%/50%/75% are three different unlabeled rates.

**Table 7:** Classification accuracy of M-training with 75%-unlabeled rate (%).

	Training Data Set		Test Data Set		
	Classification Accuracy (Initial)	Classification Accuracy (Final)	Classification Accuracy (Initial)	Classification Accuracy (Final)	Impro
Benzene	100	100	44.44	72.22	62.51
Toluene	100	100	43.75	46.88	7.15
Formaldehyde	100	100	75	95.31	27.08
All-3	100	100	59.09	80.3	35.89

**Table 8:** Classification accuracy of M-training with 50%-unlabeled rate (%).

	Training Data Set		Test Data Set		
	Classification Accuracy (Initial)	Classification Accuracy (Final)	Classification Accuracy (Initial)	Classification Accuracy (Final)	Impro
Benzene	100	100	50	88.89	77.78
Toluene	100	100	81.25	100	23.08
Formaldehyde	100	100	84.78	100	17.95
All-3	100	100	74.24	96.97	30.62

**Table 9:** Classification accuracy of M-training with 25%-unlabeled rate (%).

	Training Data Set		Test Data Set		
	Classification Accuracy (Initial)	Classification Accuracy (Final)	Classification Accuracy (Initial)	Classification Accuracy (Final)	Impro
Benzene	100	100	69.44	100	44.01
Toluene	100	100	81.25	100	23.08
Formaldehyde	100	100	82.81	92.19	11.33
All-3	100	100	78.79	96.21	22.11

**Table 10:** Amount of samples in each *c_i_* of M-training with different unlabeled rates.

	0.25		0.5		0.75	
	Initial	Final	Initial	Final	Initial	Final
*c_1_*	223	322 (99)	149	603 (454)	74	246 (172)
*c_2_*	223	322 (99)	149	214 (65)	74	354 (280)
*c_3_*	223	322 (99)	149	407 (258)	74	236 (162)
*c_4_*	223	223 (0)	149	153 (4)	74	74 (0)

Note: 322 (99) means there are 322 samples in the training data set of *c_1_*, and 99 samples more than its initial training data set (223).
